# Evaluating Glycemic Control During Basalin or Lantus Administration in Adults With Controlled Type 2 Diabetes Mellitus Using Continuous Glucose Monitoring

**DOI:** 10.3389/fendo.2021.754820

**Published:** 2021-11-30

**Authors:** Huiying Wang, Yunting Zhou, Xiaofang Zhai, Bo Ding, Ting Jing, Xiaofei Su, Huiqin Li, Jianhua Ma

**Affiliations:** Department of Endocrinology, Nanjing First Hospital, Nanjing Medical University, Nanjing, China

**Keywords:** insulin glargine, continuous glucose monitoring system, glycemic variability, Basalin, Lantus

## Abstract

**Aim:**

This study aims at evaluating glycemic control during Basalin or Lantus administration in adults with controlled type 2 diabetes mellitus using continuous glucose monitoring system (CGM).

**Methods:**

47 patients with well-controlled T2DM using both Basalin and oral hypoglycemic drugs were recruited. CGM were applied from day 1 to day 3 with the unchanged dose of Basalin and then removed from day 4. A washout was performed with Lantus at the same dose as Basalin from day 4 to day 10. Then patients were continued to install the CGM under Lantus administration from day 11 to day 13. Variables of CGM, such as the area under the curve (AUC) for both hyperglycemia and hypoglycemia, 24h mean blood glucose (24h MBG), 24h standard deviation of blood glucose (24h SDBG), 24h mean amplitude of glycemic excursion (24h MAGE), PT (percentage of time), and time in range (TIR), were calculated and compared between Basalin group and Lantus group.

**Results:**

The group of Lantus showed lower 24h MBG (p<0.01), 24h MAGE (p<0.05), and lower 24h SDBG (p<0.01) than the Basalin group. Lantus−treated patients had a lower PT and AUC when the cut-off point for blood glucose was 10 mmol/L (p<0.05) and 13.9 mmol/L (p<0.05), respectively. In this study, no patient developed symptomatic hypoglycemia, few hypoglycemia was observed and there was no difference of hypoglycemia between the two groups.

**Conclusion:**

In patients with well-controlled T2DM who were treated with insulin glargine, Lantus group showed lower MBG, GV, and lower PT (BG > 10.0 mmol/L, BG > 13.9 mmol/L) than Basalin group. In summary, for T2DM population with HbA1c ≤ 7%, Lantus may be a better choice compared with Basalin.

## Introduction

Recent aging of the Chinese population and lifestyle changes have increased the prevalence of diabetes to endemic levels (1). The prevalence of diabetes in China has increased from 0.67% in 1980 to 12.8% in 2017 (2). The high incidence of type 2 diabetes mellitus (T2DM) and its severe complications are a great burden to the government, health care systems, society, and individuals (3).

High glycemic variability (GV) is associated with various complications of T2DM. Some studies suggested that GV was an independent risk factor of micro- and macrovascular complications of diabetes (4–6). Del García et al. found that GV was associated with oxidative stress and other complications of diabetes (7, 8). Other studies demonstrated GV was an independent poor prognostic factor for patients with acute coronary syndrome (9). Furthermore, in a meta-analysis, it was suggested that high GV may cause cardiovascular disease. Controlling GV could improve insulin sensitivity, reduce the thickness of the intima of the carotid artery, and decrease the risk of cardiovascular disease (10). Access to the devices of continuous glucose monitoring (CGM) has increased worldwide, T2DM care has been greatly modified (11). With the increasing available knowledge on GV, reducing the incidence of extreme glucose excursions is the key to T2DM therapy (12). Benalia M et al. found that mean amplitude of glycemic excursion (MAGE) was one of the reference indicators for measuring GV (13).

Basal insulin therapy is a supplementary treatment for diabetes. It is widely used in clinic and recognized as the recommended molecule for initial insulin therapy in China (9). Insulin glargine is a long-acting insulin analog made using recombinant DNA technology, with greater predictability, and causes fewer hypoglycemia episodes, especially at night. On the base of human insulin, insulin glargine used glycine to replace aspartic acid at position 21 at the C end of A chain, and adds two amino acids-arginine at the C end of B chain. Glycine with neutral charge at the C end of A chain prevents the formation of deamination products and makes insulin glargine stable. The recombined insulin analogue can be prepared into a stable and clear injection under partial acid conditions (pH value is 4.0). Since the pH value (7.4) of tissue fluid is close to the isoelectric point of insulin glargine, the solubility of insulin glargine is reduced after subcutaneous injection, forming small precipitation, which is slowly dissolved into monomers and released into the blood. Its 24-hour insulin level is similar with the basal insulin secreted by normal people, which can play a stable and lasting role.

Basalin is a recombinant insulin analog produced by Gan Li Pharmaceutical Co., Ltd. in China and widely used for the lower price. It has the same pharmacodynamics and pharmacokinetic parameters as Lantus. Since 2000, Lantus, a long-acting basal insulin analog produced by a French company, Sanofi, is leading in the Chinese market because of its convenient once-a-day administration routine and the absence of any related peak activity. Lantus and Basalin are both long-acting, human insulin analogue with the same molecular weight and insulin chain. Apart from the primary structure, the secondary and tertiary structures of Lantus and Basalin are quite different. Basalin was the first biosimilar of insulin glargine marketed in China, and has become a commonly used treatment option for many Chinese physicians. However, while biosimilars are developed to be highly similar with originator therapies, due to the degree of natural variability inherent to biological drugs, which are produced using living organisms, and because of differences in manufacturing processes between products, they are not identical to the original therapy. Most studies of insulin glargine in the world are limited to Lantus, and the effectiveness and safety of Lantus have been fully verified. There are few studies on the comparison between Basalin and Lantus, but Basalin has higher cost performance for its lower price. Our previous study demonstrated that Basalin and Lantus had a similar mean blood glucose (MBG) (14); however, among patients with HbA1c ≤ 7%, the MBG in the Lantus group tended to be lower than that in the Basalin group. Thus this study was conducted to investigate the difference in glycemic control between Basalin and Lantus in patients with well-controlled T2DM (HbA1c ≤ 7%).

## Methods

### Patients

This prospective, self-controlled, single-center clinical trial was approved by the Ethics Committee of Nanjing First Hospital affiliated to Nanjing Medical University. (No. NCT040746). 50 outpatients with T2DM (HbA1c ≤ 7%) who used Basalin and oral drugs more than 3 months were recruited between February 2018 and May 2019, and 47 patients completed the trial and were included in the analysis.

The inclusion criteria were as follows: 1) patients diagnosed with T2DM as defined by the World Health Organization in 1999, 2) HbA1c ≤ 7%, 3) patients aged ≥ 18 years, 4) a body mass index (BMI) between 19 and 35 kg/m^2^, 5) patients using both glargine and oral hypoglycemic drugs (patients who used constant glargine doses [daily dose >0.2 IU/kg/day] for more than 1 month), and 6) patients with relatively constant diet and exercise habits. The exclusion criteria were as follows: 1) patients with type 1 diabetes mellitus; 2) patients with serious acute and/or chronic complications, including ketoacidosis or hyperosmolar state, end-stage renal disease, and severe cardiovascular diseases; 3) patients with severe infectious diseases; 4) patients with known cancers; and 5) patients with cognitive disorders, drug abuse, or alcoholism. The sample size of patients was calculated using the standard formula of paired t-test for dependent samples on both sides with the pass 11.0 software. The power and significance level were 80% and 5%, respectively.

### Treatment Protocol

50 patients have been informed and signed consents. General information (such as age, sex, duration of T2DM, types and dosage of oral hypoglycemic medication, insulin type and dosage, and disease complications) of the patients was collected by a fixed researcher. BMI was calculated as the weight divided by the square of height (kg/m^2^). Blood samples from all patients were collected after fasting overnight (> 10 h). HbA1c was measured using a high-performance liquid chromatography assay (Bio-Rad Laboratories, Inc. CA, USA).

**Figure 1:**
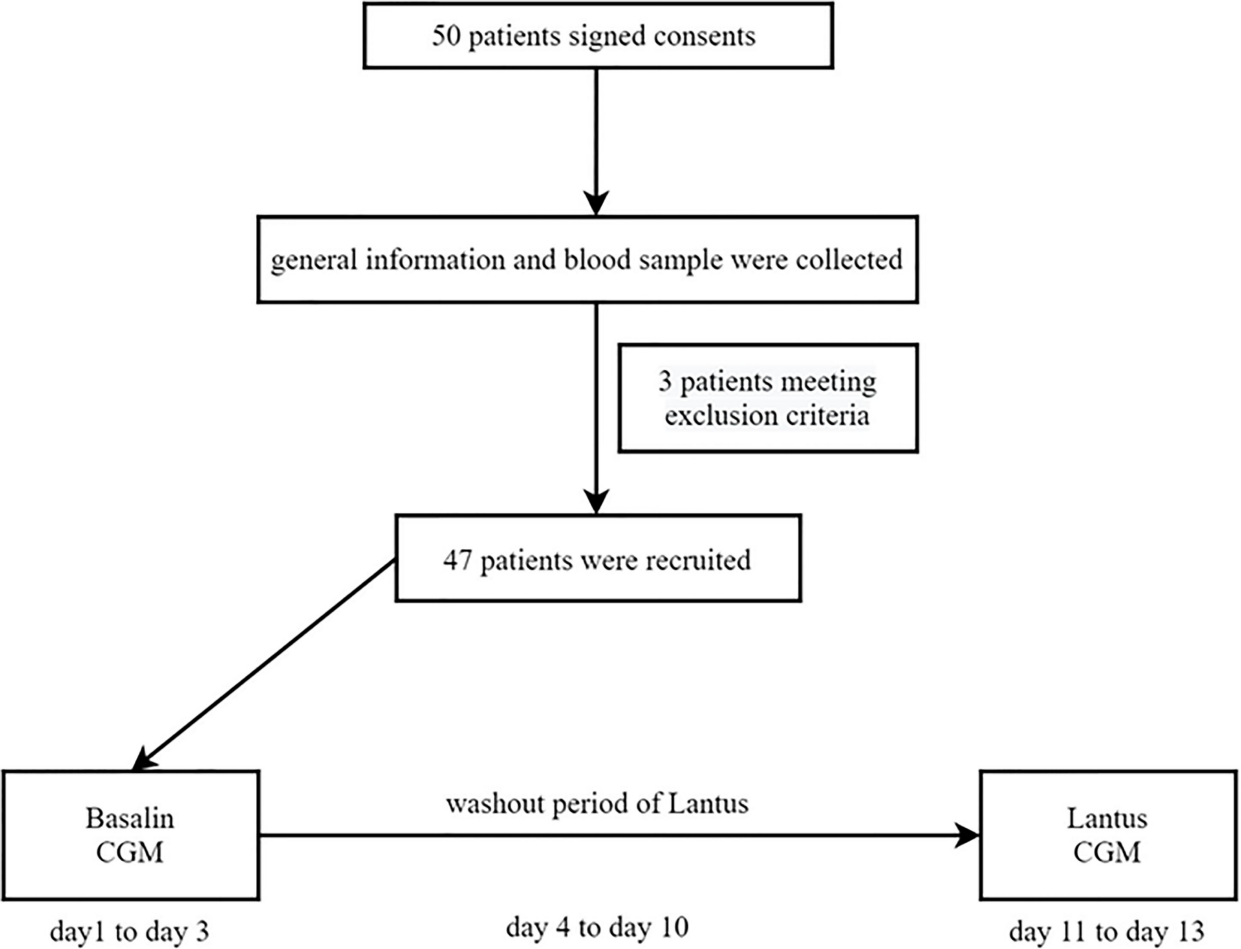
Flow chart.

47 patients who meet the criteria with well-controlled T2DM using both Basalin and oral hypoglycemic drugs were recruited. CGM were applied from day 1 to day 3 with the unchanged dose of Basalin and then removed from day 4. A washout was performed with Lantus at the same dose as Basalin from day 4 to day 10. Then patients were continued to install the CGM under Lantus administration from day 11 to day 13. The flow chart was shown in **Figure 1**.

Variables of CGM were collected for further analysis. CGM was installed by endocrine specialist nurses. Patients were blind to the type of insulin and the data of CGM before the study was completed.

CGM was calibrated to measure peripheral glycemia levels four times daily. Recording any incidences of hypoglycemia (blood glucose level < 3.9 mmol/L), allergic reactions, and other abnormalities. Patients would be advised to eat sugars if they occurred hypoglycemic symptoms. The types and doses of oral drugs during the study were consistent with those before the study. The patients maintained constant diet and exercise habits during the study period. Data was collected from 00:00 to 24:00 on the second day of CGM. The following parameters were calculated using the CGM data every 24h: 1) 24h MBG and 24h SDBG; 2) 24h MAGE: the mean fluctuation amplitude value from peak to valley every 24h; 3) Percentages of time (PT) and area under the curve (AUC) for hyperglycemia with cut-off points of 10 mmol/L and 13.9 mmol/L respectively; 4) PT and AUC for hypoglycemia with cut-off points of 2.8 mmol/L (15), 3.9 mmol/L, and 4.4 mmol/L (16) respectively; 5) Time in range (TIR): glycemia levels ranging from 3.9 mmol/L to 10 mmol/L.

### Statistical Analysis

All data were recorded and exported from the CGMS 3.0 (Medtronic Mini Med, USA) software analysis system. Continuous variables are shown as mean ± standard deviation (SD), and non-normally distributed data are presented as median (interquartile range). MBG, TIR, MAGE and SD of Basalin group and Lantus group are normally distributed data and shown as mean ± SD. The difference of MBG, TIR, MAGE and SD between Basalin group and Lantus group were examined using Student’s t test. PT and AUC are non-normally distributed data and presented as median (interquartile range). The non-parametric ANOVA was used to compare PT and AUC. Data were analyzed using SPSS Statistics package version 21. The accepted level of significance was 0.05.

## Results

### Demography and Clinical Data

Among 47 patients, 29 patients are male and 18 patients are females. The average age was 58.1 ± 10.1 years. The average BMI was 25.0 ± 3.0 kg/m^2^ and the duration of T2DM was 9.6 ± 5.8 years. The dose of insulin was 0.26 ± 0.09 U/kg/day and the HbA1c was 6.58 ± 0.41. The duration of glargine treatment was 3.54 ± 3.41 years (**Table 1**). Oral drugs used throughout the study, including metformin, α-glucosidase inhibitor as well as insulin secretagogues, were shown in **Table 2**.

**Table 1:** Patients’ baseline characteristics.

Case (n)	47
Sex (M/F)	29/18
Age (years)	58.1 ± 10.1
BMI (Kg/m^2^)	25.0 ± 3.0
Duration of T2DM (years)	9.6 ± 5.8
Insulin dose (IU/kg/day)	0.26 ± 0.09
HbA1c	6.58 ± 0.41
Duration of glargine (years)	3.54 ± 3.41

Data are normally distributed data and presented as the mean ± SD, M, male; F, female; BMI, Body mass index; HbA1c, glycated hemoglobin.

**Table 2:** Drugs used in addition to basal insulin.

Case (n)	47
metformin	34
α-glucosidase inhibitor	23
insulin secretagogues	27
DPP-4 inhibitor	4
kinds of oral anti-diabetic drugs (1/2/3)	14/25/8

DPP-4 inhibitor dipeptidyl peptidase-4 inhibitor.

### CGMS Glucose Profile

**Figure 2:**
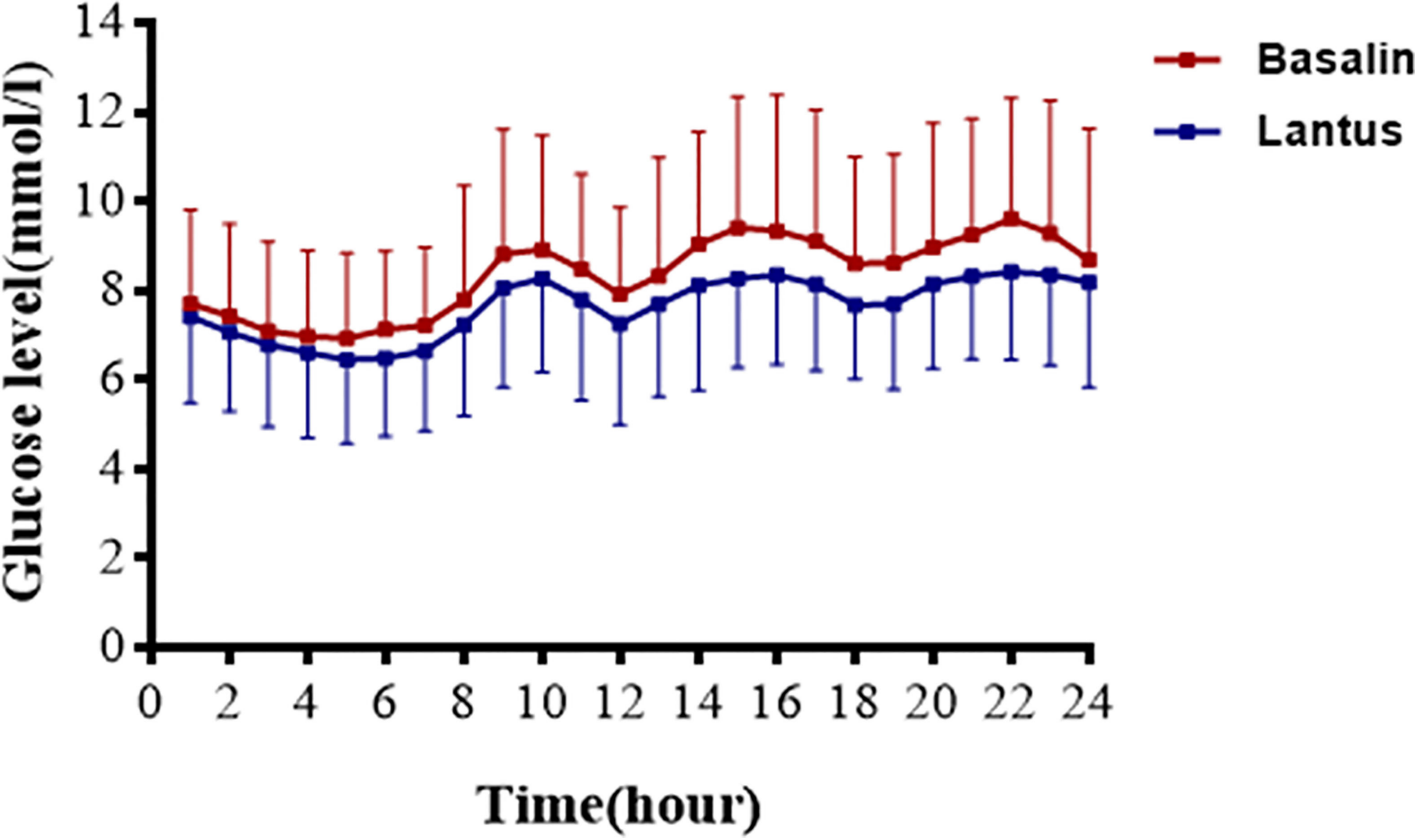
Graph present the glucose profiles of Basalin group and Lantus group.

The 24h CGMS glucose profile of patients injected with Lantus or Basalin were shown in **Figure 2**. During the treatment period, the 24h MBG of Lantus was lower than that of Basalin. The 6-12h MBG, 12-18h MBG and 18-24h MBG were higher in the Basalin group than that in the Lantus group (**Table 3**). The MBG before lunch and dinner were both higher in the Basalin group than that in the Lantus group (**Table 4**).

**Table 3:** 6-h MBG.

Glargine (47)	0–6 h (mmol/L)	6–12 h (mmol/L)	12–18 h (mmol/L)	18–24 h (mmol/L)
Basalin	7.20 ± 1.82	8.19 ± 1.59	8.97 ± 2.24	9.07 ± 2.02
Lantus	6.80 ± 1.67	7.54 ± 1.59	8.04 ± 1.52	8.19 ± 1.64
P	0.21	<0.01**	<0.01**	0.01**

The 6-h MBG are normally distributed data and shown as mean ± SD. **P ≤ 0.01, the difference of MBG between Basalin group and Lantus group were examined using Student’s t test.

**Table 4:** Mean 1-h pre-prandial blood glucose levels.

Glargine (47)	1 h before breakfast MBG (mmol/L)	1 h before lunch MBG (mmol/L)	1 h before dinner MBG (mmol/L)
Basalin	7.21 ± 1.76	8.47 ± 2.14	9.11 ± 2.93
Lantus	6.63 ± 1.79	7.80 ± 2.27	8.14 ± 1.94
P	0.05	0.04*	0.02*

The mean 1-h pre-prandial blood glucose levels are normally distributed data and shown as mean ± SD. *P < 0.05, the difference of MBG between Basalin group and Lantus group were examined using Student’s t test.

### Variables of CGM

Treatment with Lantus showed lower 24h MBG, the Lantus group also showed lower 24h SDBG and 24h MAGE than the Basalin group. PT (> 13.9 mmol/L, > 10 mmol/L), and AUC (BG > 13.9 mmol/L, BG > 10.0 mmol/L) of Basalin group are all higher than that of Lantus group. The TIR of the Lantus group showed a more favorable trend than that of the Basalin group, but the difference was not statistical significance (**Table 5**).

**Table 5:** The parameters were calculated using the CGM data.

		Basalin (47)	Lantus (47)	P value
TIR [3.9~10.0mmol/L)]		80% ± 17%	84% ± 17%	0.05
24h MBG (mmol/L)		8.36 ± 1.48	7.64 ± 1.28	<0.01**
24h SD (mmol/L)		1.99 ± 0.96	1.61 ± 0.69	0.01**
24h MAGE (mmol/L)		5.01 ± 2.42	3.76 ± 1.66	<0.01**
PT	>13.9mmol/L	0.00 (0.00,0.03)	0.00 (0.00,0.00)	<0.01**
	>10mmol/L	0.17 (0.03,0.28)	0.10 (0.00,0.19)	0.03*
AUC	>13.9mmol/L	0.00 (0.00,0.02)	0.00 (0.00,0.00)	<0.01**
	>10mmol/L	0.22 (0.01,0.55)	0.09 (0.00,0.23)	<0.01**
PT	<4.4mmol/L	0.00 (0.00,0.01)	0.00 (0.00,0.23)	0.30
	<3.9mmol/L	0.00 (0.00,0.00)	0.00 (0.00,0.00)	0.61
	<2.8mmol/L	0.00 (0.00,0.00)	0.00 (0.00,0.00)	0.65
AUC	<4.4mmol/L	0.00 (0.00,0.00)	0.00 (0.00,0.01)	0.42
	<3.9mmol/L	0.00 (0.00,0.00)	0.00 (0.00,0.00)	0.61
	<2.8mmol/L	0.00 (0.00,0.00)	0.00 (0.00,0.00)	0.66

TIR, time in range; MBG, mean blood glucose; SDBG, standard deviation of blood glucose; MAGE, mean amplitude of glycemic excursion; TAR, time above range; TBR, time below range; AUC, the area under the curve (*P<0.05; **P ≤ 0.01). MBG, TIR, MAGE and SD of Basalin group and Lantus group are normally distributed data and shown as mean ± SD. The difference of MBG, TIR, MAGE and SD between Basalin group and Lantus group were examined using Student’s t test. PT and AUC are non-normally distributed data and presented as median (interquartile range). The non-parametric ANOVA was used to compare PT and AUC.

### Adverse Events

There were no serious adverse reactions occurred during the study.

### Hypoglycemia

In this study, no patient developed symptomatic hypoglycemia. PT and AUC (<4.4mmol/L, <3.9mmol/L, <2.8mmol/L respectively) between Basalin group and Lantus group were compared. Few hypoglycemia was observed and there was no difference of hypoglycemia between the two groups (**Table 5**). The PT (<3.9mmol/L) of Basalin group was 0.00 (0.00, 0.00) and that of Lantus group was 0.00 (0.00, 0.00).

## Discussion

In this study, patients who had been using Basalin combined with oral hypoglycemic drugs for more than 3 months were recruited. Physical exercise is considered a cornerstone for both the prevention and treatment of T2DM, as it exerts both acute and chronic beneficial effects on insulin sensitivity (17). In this study, patients were asked to maintain the same exercise habits during the study as before recruitment.

As a supplementary treatment, basal insulin is commonly used in patients with poorly controlled T2DM receiving oral hypoglycemic agents (18, 19). Compared with medium-acting insulin, long-acting insulin analogs have greater advantages in terms of longer acting time, better fasting and pre-meal glycemic control, and a lower frequency of hypoglycemia (20). Blood glucose levels were measured using peripheral blood glucose, intravenous blood glucose, HbA1c, and CGM (21). In our previous research, we found that Basalin and Lantus had a similar MBG; however, among patients with HbA1c ≤ 7%, the MBG in the Lantus group tended to be lower than that in the Basalin group. In this study, we further compared the difference between Basalin and Lantus in adults with well-controlled T2DM (HbA1c ≤ 7%). The MBG in the Basalin and Lantus groups was respectively 8.36 ± 1.48 mmol/L and 7.64 ± 1.28 mmol/L (p<0.01). Therefore, in our study, for the patients with well-controlled T2DM, the Lantus group had more favorable outcomes, with a lower MBG, MAGE, SDBG, PT (BG > 13.9 mmol/L, BG > 10.0 mmol/L), and AUC (BG > 13.9 mmol/L, BG > 10.0 mmol/L) than those of the Basalin group. This finding is consistent with the findings in a study by Hu et al. (22).

Blood glucose fluctuation is closely related to complications of diabetes. MAGE is associated with somatomedins, such as insulin-like growth factor-1 (23). GV is an independent risk factor for diabetic complications when adjusted for mean glucose levels (24). MAGE has greater negative effects on cardiovascular disease than HbA1c (25). Although the MBG and glucose levels of the patients in this trial were well-controlled, GV remains a major issue that needs to be addressed. Since HbA1c reflects long-term average blood glucose levels, which is associated with diabetic complications. However, it is limited for GV estimation. In patients with well-controlled T2DM, Lantus had a lower MAGE than Basalin (3.76 ± 1.66 mmol/L vs. 5.01 ± 2.42 mmol/L, p<0.05). TIR is also an indicator used to assess glycemic control (26). Beck et al. found that TIR is closely related to the risk of microvascular complications and should be an acceptable endpoint in clinical trials (27). In addition, a recent study has proved that higher TIR level was independently associated with better peripheral nerve function. CGM-derived TIR may be a promising approach to screen patients for further assessment of possible diabetic peripheral neuropathy, which means that Lantus has better clinical potential than Basalin in the prevention of diabetic peripheral neuropathy (28). TIR was found to be associated with thickness of the intima and media of the carotid artery in patients with T2DM (29). The target of TIR is 70% (30). In this study, we compared the TIR between Basalin and Lantus in patients with well-controlled T2DM and found that the TIR was above 70% in both groups.

Hypoglycemia is the main side effect of exogenous insulin use (31), and in addition to acute changes in mental status (32), severe hypoglycemia can cause cognitive impairment, falls leading to fractures, and cardiac arrhythmias, resulting in sudden death. CGM has a distinct advantage of being able to detect asymptomatic hypoglycemia and nocturnal hypoglycemia (33). There was no severe hypoglycemia associated with either treatment during our study, and few hypoglycemia (PT<3.9mmol/L) was observed and there was no difference between the two groups. This study had some limitations. Firstly, limited by conditions, the CGM in this study was collected for only 3 days and the data of the second day was obtained for analysis. We will consider extending the installing period of CGM in future related research. Secondly, only patients with HbA1c ≤ 7% were involving in present investigation. Involving patients with different levels of glycemic control will provide stronger evidence of therapeutic efficacy for Lantus treatment protocol.

## Conclusions

In patients with well-controlled T2DM with insulin glargine therapy, Lantus administration group showed lower MBG, GV, and lower PT (BG > 10.0 mmol/L, BG > 13.9 mmol/L) than those in Basalin administration group. Few hypoglycemia was observed and there was no statistical difference of hypoglycemia between the two groups. In summary, for T2DM population with HbA1c ≤ 7%, Lantus may be a better choice for glycemic control compared with Basalin.

## Data Availability Statement

The raw data supporting the conclusions of this article will be made available by the authors, without undue reservation.

## Ethics Statement 

This study was registered in ClinicalTrials.gov identifier (NCT04074603) and was approved by the Ethics Committee of Nanjing First Hospital, Nanjing Medica l Univerity. The patients/participants provided their written informed consent to participate in this study.

## Author Contributions

HW and YZ analyzed data and wrote the manuscript. XZ, BD, and TJ performed the experiments and collected samples. XS modified the manuscript. HL and JM conceived, designed and directed the study. All authors contributed to the article and approved the submitted version.

## Funding

This study was partly supported by the National Key R&D Program of China (No. 2018YFC1314103), the National Natural Science Foundation of China (No. 81870563), and Jiangsu Provincial Double Innovation Doctor Program (No. JSSCBS20211546, to YZ)

## Conflict of Interest

The authors declare that the research was conducted in the absence of any commercial or financial relationships that could be construed as a potential conflict of interest.

## Publisher’s Note

All claims expressed in this article are solely those of the authors and do not necessarily represent those of their affiliated organizations, or those of the publisher, the editors and the reviewers. Any product that may be evaluated in this article, or claim that may be made by its manufacturer, is not guaranteed or endorsed by the publisher.
